# Supplementary material for: Drug target prediction through deep learning functional representation of gene signatures
Source: Nat Commun. 2024 Feb 29;15:1853. doi: 10.1038/s41467-024-46089-y (PMC10904399; doi:10.1038/s41467-024-46089-y)
Supplement: Supplementary file 1 — Supplementary Information [file 41467_2024_46089_MOESM1_ESM.pdf]

Supplementary Information

Drug target prediction through deep learning functional representation of gene signatures

Chen et al.

Supplementary Notes

Supplementary Note 1: Gene signature embeddings derived from other gene embedding methods

Gene signature embeddings were also derived from other gene embedding methods, including OPA2Vec[1], Gene2vec[2], and clusDCA[3], to compare with FRoGS.

OPA2Vec applies the word2vec[4] model to jointly generate embeddings for genes and GO terms. For this, it first generates sentences from GO annotations and GO metadata to form a corpus, in which genes and GO terms are treated as words. The PubMed[5] abstracts are used as a corpus by OPA2Vec for pretraining. Using OPA2Vec's gene embeddings, an input gene set can be represented by averaging embeddings of individual genes in the set, denoted as 'OPA2Vec Gene'. For completeness, we also generate gene set embeddings using OPA2Vec's GO embeddings, by first adding up embeddings of GO terms associated with each gene as the gene embedding and then averaging gene embeddings in the set, denoted as 'OPA2Vec GO'. The gene and GO embeddings used for comparison were generated using the provided code and data in the OPA2Vec GitHub repository (https://github.com/bio-ontology-research-group/opa2vec).

Gene2vec generates gene embeddings from transcriptome-wide gene co-expression networks derived from large-scale Gene Expression Omnibus datasets. It uses a Skip-Gram model to learn gene embeddings from highly co-expressed gene pairs. The pretrained gene embeddings obtained from the Gene2vec GitHub repository (https://github.com/jingcheng-du/Gene2vec) were used for comparison.

clusDCA generates embeddings of GO terms by running random walks on the directed acyclic graph structures of GO hierarchies and then applying matrix factorization. The provided code and data from the clusDCA GitHub repository (https://github.com/wangshenguiuc/clusDCA) were used to generate the clusDCA GO embedding. Gene embeddings of Gene2vec and GO embeddings of clusDCA were used to generate gene signature embeddings using the same methods as described above.

While OPA2Vec takes into account GO annotations of genes which are treated as sentences, it does not explicitly model the co-functions of genes and the co-occurrence of GO terms. In comparison, this information can be reflected in our GO hypergraph. Gene2vec embeds genes solely based on gene expression data, overlooking the essential GO annotations that directly reflect gene functions. On the other hand, clusDCA embeds individual GO terms but does not consider each gene as a union of multiple functions. These differences might explain why FRoGS, in its modeling of gene functions, demonstrates significantly improved performance.

Supplementary Note 2: The influence of data sampling strategy on model performance

Zhong et al.[6] recently published a Siamese spectral-based graph convolutional network (SSGCN) model by leveraging a protein-protein interaction network contained by transcriptional signatures. With 123 drugs and the top 100 predicted candidates, SSGCN showed an improved target recall of 0.71-0.84

compared to 0.15 using CMap[6]. For SSGCN model training, positive compound-target pairs were based on the Broad Institute's compound annotation[7], where known compound targets were highly concentrated in functional categories related to kinase signaling and cancer-related processes (Supplementary Fig. 2). In contrast, negative compound-target pairs in the dataset were generated by pairing compounds of known targets with genes randomly selected from the whole genome, where no functional enrichment should be expected. Therefore, gene members of highly enriched pathways such as oncogenic kinases occurred much more frequently in the positive pairs than in the negative pairs of both training and test datasets. Compound-target predictions should conceptually be solely based on the interaction terms between compound features and target features (in the forms of shRNA/cDNA signatures). However, the strong association between targets and the positive outcome inherited from the described sampling strategy raises the concern that a model might make good predictions heavily relying on target features alone and is therefore biased towards targets that frequently appeared in the positive pairs of training data, which could have resulted in an overestimated model performance but not generalizable in real applications.

To investigate this possibility, we retrained our FRoGS based deep learning model using the same data split tactic as used in SSGCN and followed its data generation strategy. Using the SSGCN's evaluation strategy, our new model achieved a high top-30 accuracy of 0.59 and a top-100 accuracy of 0.80, which is higher than the reported performance of SSGCN and the other methods compared in its original publication (Supplementary Fig. 3). Compared to Model L's recall of 0.36, the significant boost in model recall here was purely due to the difference in how the negative training dataset was constructed. We then randomly permuted the gene identity in the query compound gene signatures, i.e., erased biological information within the query compound signatures. We expected the new model to completely fail, as any predictions would have to be based on the shRNA/cDNA gene signatures alone, given the input compound signature was nonsense. Surprisingly, the new model still achieved a high top-30 accuracy of 0.46 compared to 0.008 of random predictions (Supplementary Fig. 3). This suggests the training set constructed by SSGCN lowered the barrier for the prediction challenge, which might have resulted in a model paying an inappropriate amount of attention to target signature input alone and thus may not generalize well for real applications. In our Model L training, each target occurred in equal frequency in both our positive and negative datasets, therefore, our model had to make predictions relying solely on compound-target interactions. In addition, considering 123 drugs were used in SSGCN compared to 1438 compounds used in our recall benchmark, the different evaluation datasets might partly explain the significantly higher performance of the CMap method (16%) quoted in Zhong et al. compared to the 9.6% we observed[6]. Incorporating all the above observations, we believe the prediction of compound targets based on transcriptional signatures remains an important unsolved challenge and that successful models reported in the literature may warrant further evaluation.

Supplementary Note 3: Co-target probability based on structure similarities

Madhukar et al. previously demonstrated that structure similarity $s(c, c')$ between a query c and a reference c' served as the strongest evidence for compound target prediction according to the structure-activity relationship (SAR)[8]. A Model S_{SAR} was derived based on an in-house medicinal chemistry activity database, where it mapped s, measured by ECFP fingerprints[9], into a probability of the compound pair being co-active within the same biological assay. According to Model S_{SAR}, compound pairs with $s \geq 0.64$ have ≥ 0.8 probability to be co-active in the same assay. However, as we are not interested in predicting targets for compounds that are only structurally similar to known reference structures, Model S_{SAR} offers little value in predicting novel targets. Therefore, Model S_{SAR} is not used for target prediction in our study, but only serves to validate predictions generated by other models.

Supplementary Note 4: Construction of the pQSAR-based model

Pharmacological activities are useful for target prediction in the knowledge-based approaches[8], relying on the guilt-by-association (GBA) principle, which assumes compound pairs sharing similar activity patterns across a large assay panel tend to interact with the same targets or perturb the same underlying pathway[10]. Our company, Novartis International AG, has accumulated a large proprietary compound activity matrix consisting of 6 million chemical structures and more than 12,000 biological assays (see Methods), where assays cover a diverse collection of gene targets including kinases, proteases, transcription regulators, G-protein-coupled receptors, nuclear hormone receptors, ion channels, and other common drug targets. Martin et al. demonstrated the missing elements in this matrix could be imputed effectively with a profile-quantitative structure-activity relationship (pQSAR) algorithm, where the Pearson r^2 between experimental pIC_{50} values and their predictions were above 0.3 for 72% of the assays[11]. This pharmacological activity matrix, hereafter referred to as the pQSAR dataset, enabled us to train an LR-based Model pQSAR, also abbreviated as Model Q, to map the correlation coefficient between a Broad compound pair's activity profile, q, into their probability of sharing the same target. The compound pair included a query compound c and a reference compound c' of a known target g, therefore, Model Q predicted the probability of a (c, g) association through $q(c, c')$. Many previous activity-based studies were based on the NCI_{60} growth inhibition screen dataset[12], and our test results showed a similar LR model only based on NCI_{60} achieved a weak Matthews correlation coefficient (MCC)[13] of 0.04 and a recall of 0.16, while Model Q based on the much larger pQSAR matrix offered a significant advantage with an MCC of 0.33 and a recall of 0.70. This outcome was expected as pQSAR matrix covered a significantly larger chemical and biological space, therefore, activity correlations across 4420 assays (1003 unique gene targets) should have been a much stronger predictor for co-targeting than assays limited to 60 NCI cancer cell lines. The performance metric of all models tested in this study are provided in Supplementary Table 1.

Supplementary Note 5: Transcriptional, pharmacological, and structural data types are orthogonal

Supplementary Fig. 4a suggests Model L does not rely on structure similarity information ($r^2 = 0.00$). Supplementary Fig. 4b illustrates there exists a correlation between q and s, especially for $s \geq 0.35$. We compute q in the pQSAR data source only for compound pairs with $s < 0.35$, therefore, limiting the correlation to a lower r^2 value of 0.10. Based on our analysis using in-house data, a similarity threshold of 0.35 corresponds to a weak 55% probability of preserving the structure-activity relationship (SAR) (Supplementary Note 3). Scatter plots in Supplementary Fig. 4c confirmed the previous study that gene signature evidence r and activity correlation q do not linearly correlate with each other.

As none of Model L, Model Q, and Model LQ (the combined model described in Supplementary Note 7) introduced in this study relied on structure similarity evidence, structure similarity can be used as an independent validation for model predictions. This also suggests the performance of these three models is expected to be generalizable for future applications for orphan compounds. Note that compound pairs with $s \geq 0.35$ were ignored when they were used as inputs for Model Q and Model LQ for the purpose of target prediction; however, when we use Model Q in the context of validating and annotating predictions from Model L and Model LQ, we consider all compound pairs regardless of their s values and we call such a model as Model *Best_Q*.

Supplementary Note 6: The complementary roles of Model *L* and Model *pQSAR*

While Model Q outperforms Model L, which is consistent with previous findings[8,14] (Supplementary Fig. 5a), the two models likely have their own strength depending on the biological context of the target. The

overlap of compounds and gene targets predictable by Model *L* and Model *Q* separately are shown in Supplementary Fig. 5b and Supplementary Fig. 5c, respectively. Only predictions for the top 5% target candidates with a probability higher than 0.8 were retained for Model *L*. Since the L1000 *r* scores originated from normalized rank scores, 0.8 probability score translates to top 1%; the more stringent threshold partially explains the relatively smaller gene counts associated with Model *L* compared to Model *Q*. Enrichment analyses were carried out with Metascape[15] on the known targets recalled by either models, which suggested both models are capable of recovering targets involved in a broad range of pathways and biological processes (Supplementary Fig. 5d, Supplementary Fig. 6a). More importantly, Model *L* predicts targets that are not recoverable by the Model *Q* (Supplementary Fig. 5e, Supplementary Fig. 6b). These model-specific pathways tend to have less significant *p*-values than those observed when both models provide similar predictions (Supplementary Fig. 5d, Supplementary Fig. 6a). Likely, each approach possesses a unique advantage inherent within the data sources, such as transcriptional response for Model *L* and enrichment of target classes covered by in-house assays and focused compound screening libraries used for Model *Q*.

Supplementary Note 7: Choose logistic regression for Model *LQ*

In this study, we adopted logistic regression models to convert features learned from a single or multiple data sources into a compound-target association probability score. Madhukar et al. combined multiple lines of evidence using a naive Bayesian model by assuming underlying features were independent of each other[8]. The logistic regression model can also be viewed as a general form of the Naive Bayesian model. On one hand, LR models translate a missing value into a 0.5 probability suggesting neither a favorable nor unfavorable outcome, which is equivalent to multiply an odd ratio of one in the Naive Bayesian model. On the other hand, logistic regression does not require features to be independent, it instead can model feature dependency to a certain extent. This is conceptually assuring, even though we do not observe significant correlation between L1000 and pQSAR datasets (Supplementary Fig. 4c).

For the standard Naive Bayesian model LQ_{NB}, the probability is modeled based on the multiplication of odd ratios:

$$p_{LQ} = \frac{1}{1+\exp\left\{-\frac{p_L}{1-p_L}\cdot\frac{p_Q}{1-p_Q}\right\}}, \tag{1}$$

where p_L and p_Q are probabilities from Model *L* and Model *Q*, respectively. We also tested a modified model $LQ_{\mathrm{NB\ clip}}$, where odd ratios less than one are ignored:

$$p_{LQ\ \mathrm{clip}} = \frac{1}{1+\exp\left\{-\max\left(1,\frac{p_L}{1-p_L}\right)\cdot\max\left(1,\frac{p_Q}{1-p_Q}\right)\right\}}. \tag{2}$$

Model *LQ* archived a recall of 0.74, surpassing both naive Bayesian models with recall of 0.67 for LQ_{NB} and 0.69 for $LQ_{\mathrm{NB\ clip}}$ (Supplementary Fig. 8, Supplementary Table 1). This suggests there remain residual dependencies between *r* and *q*, despite their negligible linear correlation coefficient.

Supplementary Note 8: Tests of viable options of combining Model *L* and Model pQSAR

We tested another non-linear model XGBoost[16], as it has become the method of choice by many Kaggle (https://www.kaggle.com) winning teams. The recall rate of XGBoost based on five-fold cross-validation

(CV) is at par with Model *LQ*. Compared to Model *LQ* with only three parameters, XGBoost is a tree-ensemble model that requires many more model parameters. With only 2340 positive samples in the training set, independent XGBoost CV model inferences have Pearson r^2 of 0.9 (Supplementary Fig. 9a) compared to 1.0 in the case of *LR* (Supplementary Fig. 9b). Conceptually compound-target pairs with higher similarity feature scores must produce higher probability output, which is guaranteed in LR models as weight parameters are positively defined. Such desirable biological interpretability is not guaranteed within more complex models such as XGBoost.

Supplementary Figures

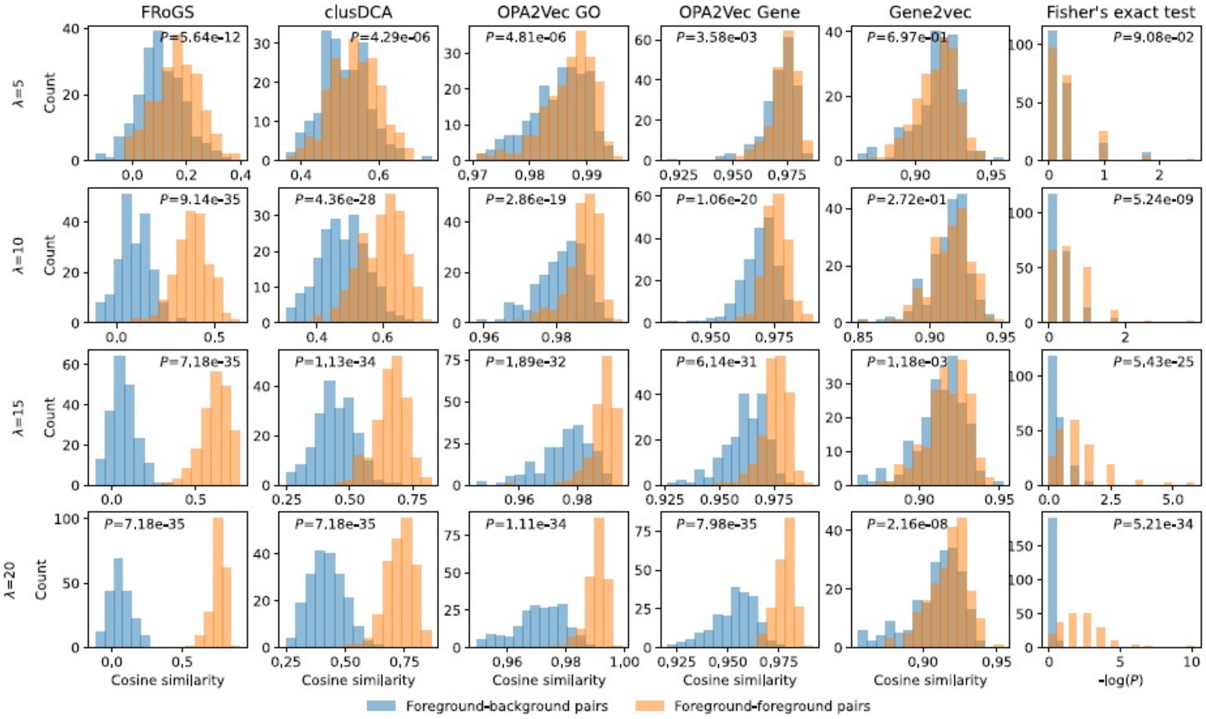

a

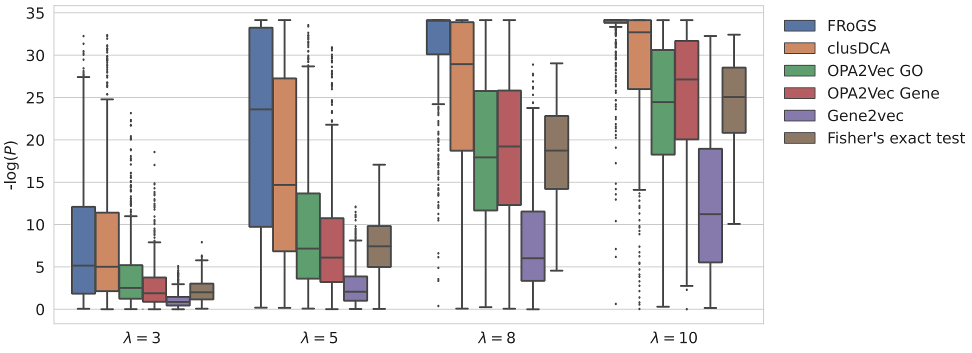

b

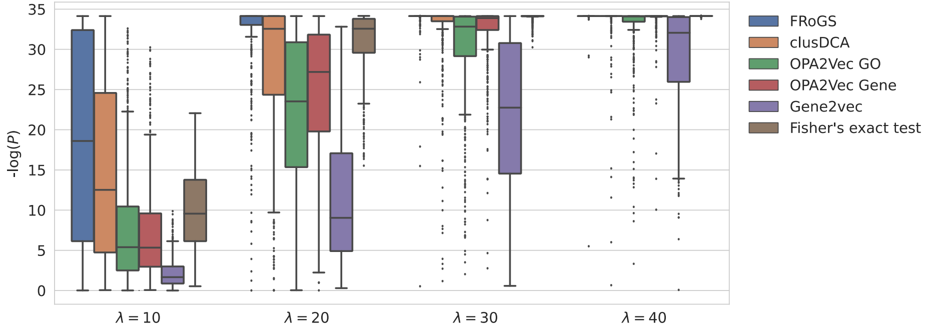

c

Supplementary Fig. 1 The differentiation of foreground and background gene signatures using simulated gene lists derived from Reactome pathways. a Distributions of the distance between two kinds of gene set pairs for different methods using pathway R-HSA-5576891 (Cardiac conduction) as an example. Algorithms attempt to distinguish pairs of foreground gene set signatures (orange) from pairs of foreground-background signatures (blue). Each row represents simulation results conducted using foreground gene sets sampled under a particular λ. Each column contains results obtained under the corresponding algorithms. When fewer pathway genes were seeded ($\lambda = 5$, the first row), the Fisher's exact test (the last column in Supplementary Fig. 1) failed to distinguish (S_{fg}, S'_{fg}) pairs from (S_{fg}, S_{bg}) pairs (p = 0.09), explaining the difficulty that current gene identity-based algorithms have when extracting weak molecular signals. FRoGS embedding (the first column in **a**), on the other hand, distinguished the two distributions with a p-value of 5.6×10^{-12}. As λ increased, the two distributions became more readily distinguishable under stronger pathway signals, and FRoGS demonstrated the best separation among all methods under comparison. **b-c** Simulation results validate that the results presented in Fig. 1c are robust against gene lists of various sizes, where the same percentages of pathway genes used in Fig. 1c (5%, 10%, 15%, and 20%) seed the foreground gene sets. **b** Results obtained with λ = 3, λ = 5, λ = 8, and λ = 10 for gene sets of size 50. **c** Results obtained with λ = 10, λ = 20, λ = 30, and λ = 40 for gene sets of size 200. Box-and-whisker plots show the median (center line), 25th, and 75th percentile (lower and upper boundary), with 1.5× inter-quartile range indicated by whiskers and outliers shown as individual data points. **a-c** one-sided Wilcoxon signed-rank test, n = 200 simulations. Source data are provided as a Source Data file.

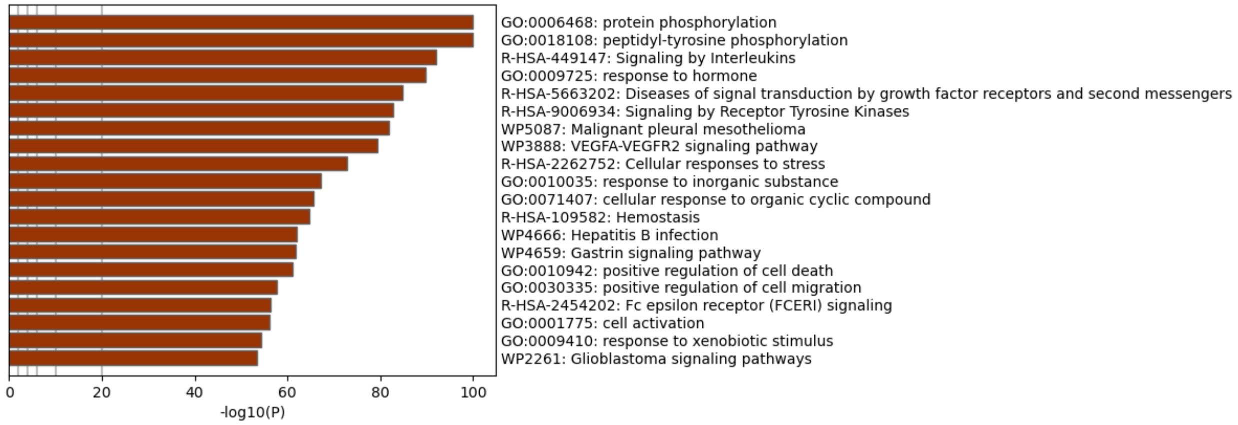

Supplementary Fig. 2 Known targets show strong functional bias. Known targets based on Broad annotations[7] are highly enriched in kinase signaling and cancer-related processes according to Metascape enrichment analysis[15]. One-sided fisher exact test, statistics including multi-test adjusted *p*-values are in source data. Source data are provided as a Source Data file.

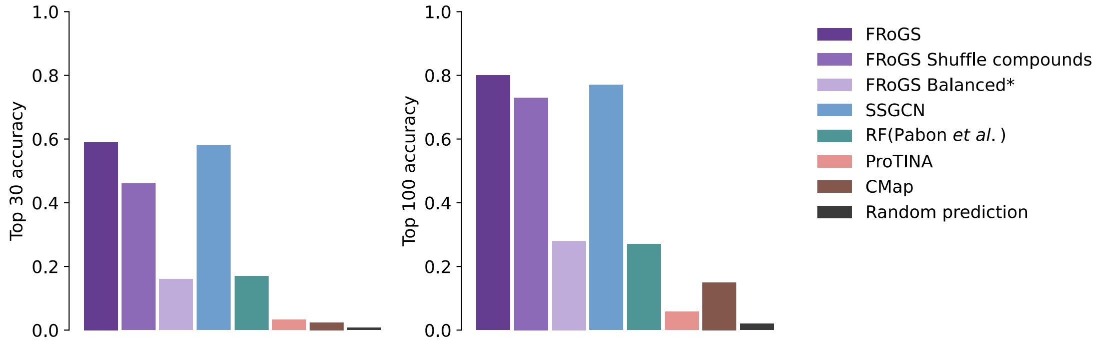

Supplementary Fig. 3 Performance comparison with other state-of-the-art compound-target prediction methods based on the SSGCN[11] dataset. The performance of FRoGS was trained on the SSGCN dataset. The performance of FRoGS Shuffle compounds was obtained by shuffling the query compound gene signatures in the input of FRoGS. FRoGS Balanced* uses the stringent data generation strategy that requires a 1:1 ratio of positive compounds to negative compounds paired with each target, which is different from the one used in SSGCN. The performance of SSGCN[6], RF (Pabon *et al.*)[17], ProTINA[18], CMap[19], and random prediction was obtained from the original publication of SSGCN. The dramatic increase in performance of our FRoGS model using the SSGCN imbalanced training dataset (comparing the 3rd bar to the 1st bar from the left) suggests the high performance of SSGCN might be partially explained by the bias introduced in the training that could limit model generalization.

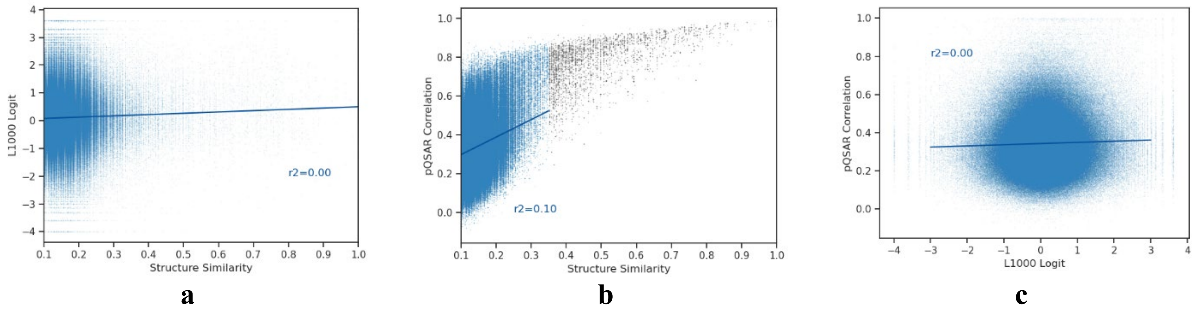

Supplementary Fig. 4 Correlation among L1000 logit score (1 - *r*)/*r*, pQSAR correlation coefficient *q*, and query-reference structural similarity score *s*. a L1000 logit and structure similarity score *s* are independent. **b** pQSAR similarity *q* and structure similarity *s* have a marginal correlation of $r^2 = 0.10$, where we only compute *q* for pairs with $s \leq 0.35$. **c** L1000 and pQSAR features are independent.

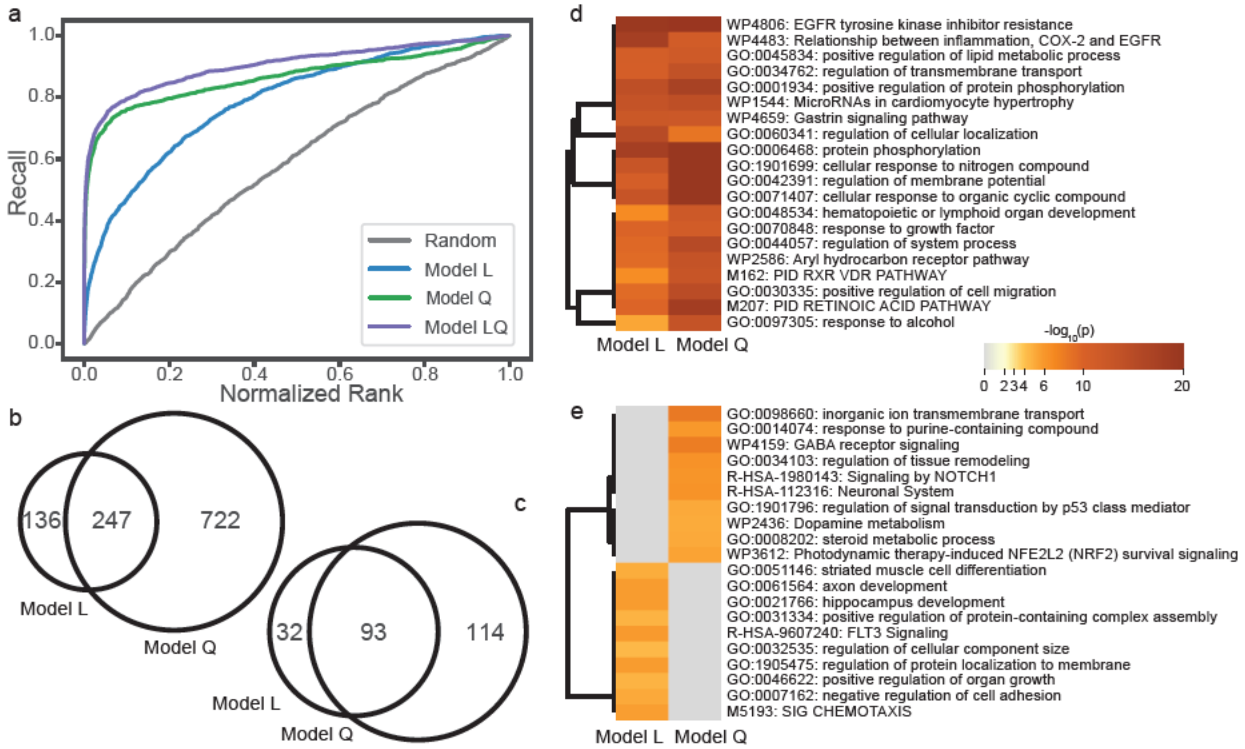

Supplementary Fig. 5 Comparison between Model *L* and Model *Q*. a Recall-rank plot for all models. **b** Venn diagram of compounds with known targets recallable with high probability ($p \geq 0.8$) by Model *L* and Model *Q*. **c** Venn diagram of known targets recallable with high probability ($p \geq 0.8$) by Model *L* and Model *Q*. **d** Top 20 commonly enriched biological process clusters based on known targets in **b**. **e** Top 20 specifically enriched biological process clusters based on known targets recalled by Model *L* and Model *Q*. **d-e** One-sided fisher exact test, statistics including multi-test adjusted *p*-values are in source data. Source data are provided as a Source Data file.

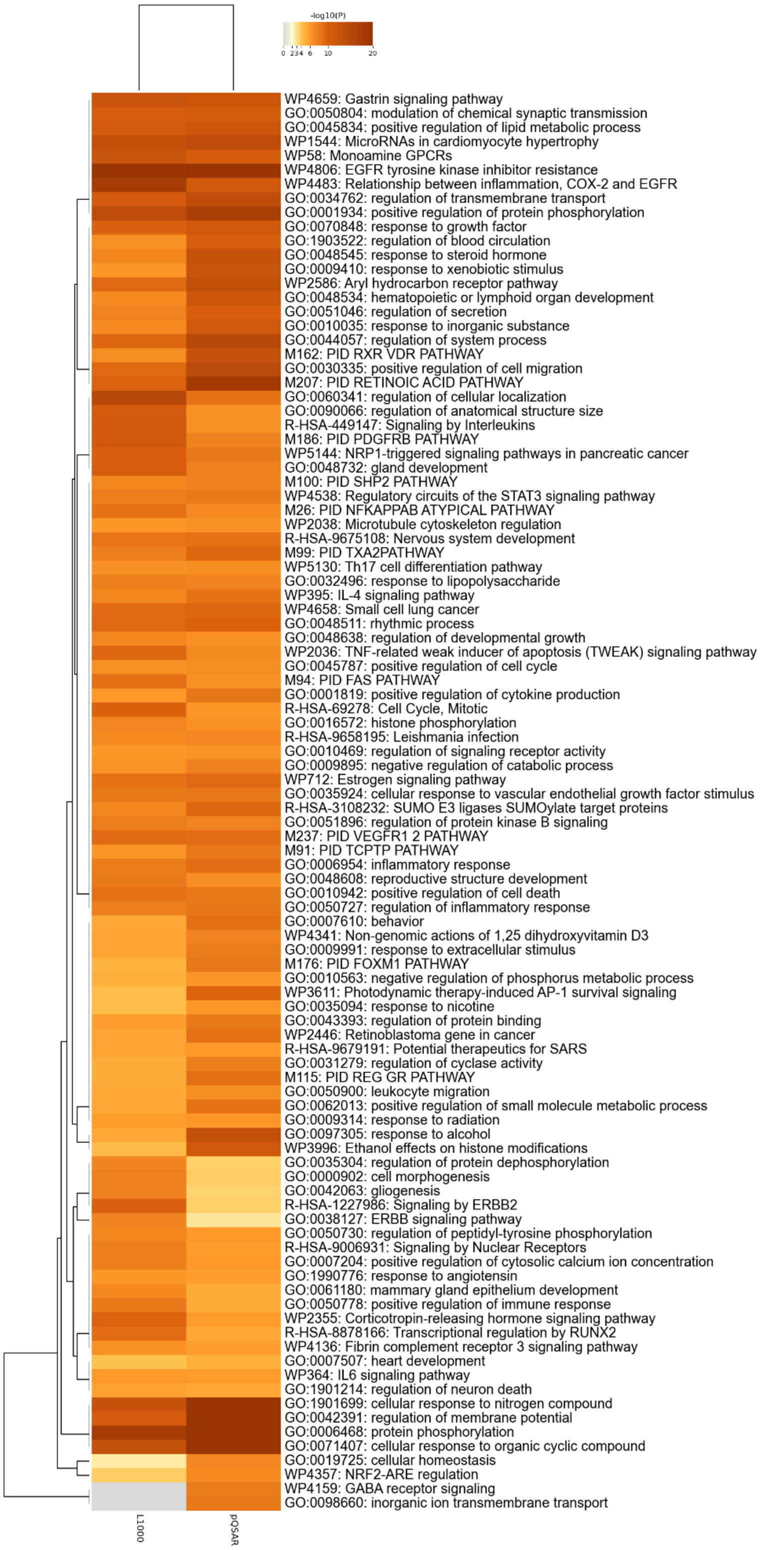
-log10(P)
0 2 3 4 6 10 20
WP4659: Gastrin signaling pathway
GO:0050804: modulation of chemical synaptic transmission
GO:0045834: positive regulation of lipid metabolic process
WP1544: MicroRNAs in cardiomyocyte hypertrophy
WP58: Monoamine GPCRs
WP4806: EGFR tyrosine kinase inhibitor resistance
WP4483: Relationship between inflammation, COX-2 and EGFR
GO:0034762: regulation of transmembrane transport
GO:0001934: positive regulation of protein phosphorylation
GO:0070848: response to growth factor
GO:1903522: regulation of blood circulation
GO:0048545: response to steroid hormone
GO:0009410: response to xenobiotic stimulus
WP2586: Aryl hydrocarbon receptor pathway
GO:0048534: hematopoietic or lymphoid organ development
GO:0051046: regulation of secretion
GO:0010035: response to inorganic substance
GO:0044057: regulation of system process
M162: PID RXR VDR PATHWAY
GO:0030335: positive regulation of cell migration
M207: PID RETINOIC ACID PATHWAY
GO:0060341: regulation of cellular localization
GO:0090066: regulation of anatomical structure size
R-HSA-449147: Signaling by Interleukins
M186: PID PDGFRB PATHWAY
WP5144: NRP1-triggered signaling pathways in pancreatic cancer
GO:0048732: gland development
M100: PID SHP2 PATHWAY
WP4538: Regulatory circuits of the STAT3 signaling pathway
M26: PID NFKAPPAB ATYPICAL PATHWAY
WP2038: Microtubule cytoskeleton regulation
R-HSA-9675108: Nervous system development
M99: PID TXA2PATHWAY
WP5130: Th17 cell differentiation pathway
GO:0032496: response to lipopolysaccharide
WP395: IL-4 signaling pathway
WP4658: Small cell lung cancer
GO:0048511: rhythmic process
GO:0048638: regulation of developmental growth
WP2036: TNF-related weak inducer of apoptosis (TWEAK) signaling pathway
GO:0045787: positive regulation of cell cycle
M94: PID FAS PATHWAY
GO:0001819: positive regulation of cytokine production
R-HSA-69278: Cell Cycle, Mitotic
GO:0016572: histone phosphorylation
R-HSA-9658195: Leishmania infection
GO:0010469: regulation of signaling receptor activity
GO:0009895: negative regulation of catabolic process
WP712: Estrogen signaling pathway
GO:0035924: cellular response to vascular endothelial growth factor stimulus
R-HSA-3108232: SUMO E3 ligases SUMOylate target proteins
GO:0051896: regulation of protein kinase B signaling
M237: PID VEGFR1 2 PATHWAY
M91: PID TCPTP PATHWAY
GO:0006954: inflammatory response
GO:0048608: reproductive structure development
GO:0010942: positive regulation of cell death
GO:0050727: regulation of inflammatory response
GO:0007610: behavior
WP4341: Non-genomic actions of 1,25 dihydroxyvitamin D3
GO:0009991: response to extracellular stimulus
M176: PID FOXM1 PATHWAY
GO:0010563: negative regulation of phosphorus metabolic process
WP3611: Photodynamic therapy-induced AP-1 survival signaling
GO:0035094: response to nicotine
GO:0043393: regulation of protein binding
WP2446: Retinoblastoma gene in cancer
R-HSA-9679191: Potential therapeutics for SARS
GO:0031279: regulation of cyclase activity
M115: PID REG GR PATHWAY
GO:0050900: leukocyte migration
GO:0062013: positive regulation of small molecule metabolic process
GO:0009314: response to radiation
GO:0097305: response to alcohol
WP3996: Ethanol effects on histone modifications
GO:0035304: regulation of protein dephosphorylation
GO:0000902: cell morphogenesis
GO:0042063: gliogenesis
R-HSA-1227986: Signaling by ERBB2
GO:0038127: ERBB signaling pathway
GO:0050730: regulation of peptidyl-tyrosine phosphorylation
R-HSA-9006931: Signaling by Nuclear Receptors
GO:0007204: positive regulation of cytosolic calcium ion concentration
GO:1990776: response to angiotensin
GO:0061180: mammary gland epithelium development
GO:0050778: positive regulation of immune response
WP2355: Corticotropin-releasing hormone signaling pathway
R-HSA-8878166: Transcriptional regulation by RUNX2
WP4136: Fibrin complement receptor 3 signaling pathway
GO:0007507: heart development
WP364: IL6 signaling pathway
GO:1901214: regulation of neuron death
GO:1901699: cellular response to nitrogen compound
GO:0042391: regulation of membrane potential
GO:0006468: protein phosphorylation
GO:0071407: cellular response to organic cyclic compound
GO:0019725: cellular homeostasis
WP4357: NRF2-ARE regulation
WP4159: GABA receptor signaling
GO:0098660: inorganic ion transmembrane transport
L1000
pQSAR

a

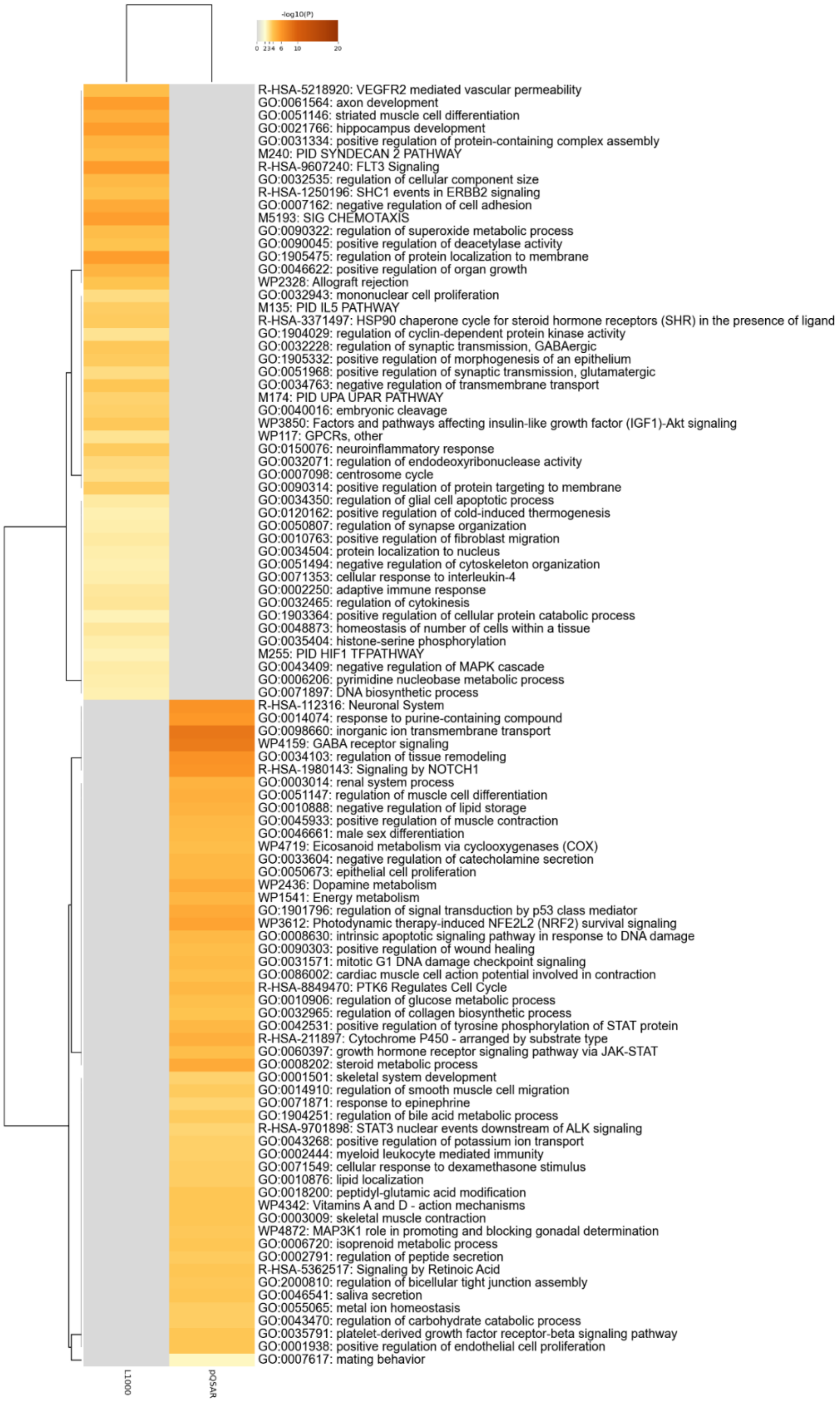

b

Supplementary Fig. 6 Top 100 enriched processes identified by Metascape[15] based on known targets recalled by Model *L* and Model *Q*. a Metascape enrichment analysis results prioritized by commonly enriched processes. **b** Metascape enrichment analysis results prioritized by specifically enriched processes. **a-b** One-sided fisher exact test, statistics including multi-test adjusted *p*-values are in source data. Source data are provided in the same Source Data files associated with Supplementary Fig. 5.d-e.

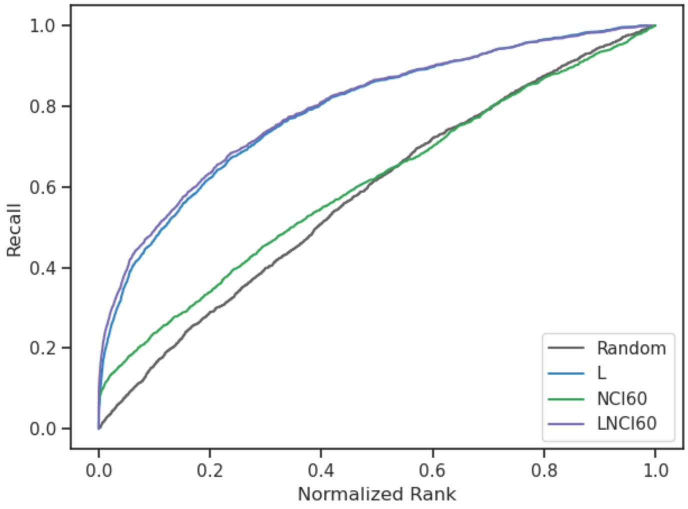

Supplementary Fig. 7 Comparison of Model *L*, Model NCI_{60}, and Model *L*+NCI_{60} (LNCI60). Recalls at different normalized ranks are shown. Source data are provided as a Source Data file.

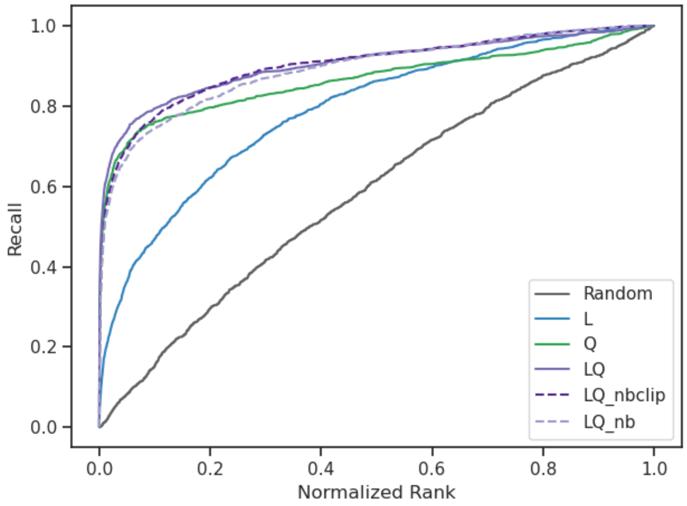

Supplementary Fig. 8 Comparison between logistic regression Model *LQ* and Naive Bayesian models LQ_{NB} and $LQ_{NB\ clip}$. Recalls at different normalized ranks are shown. Source data are provided as a Source Data file.

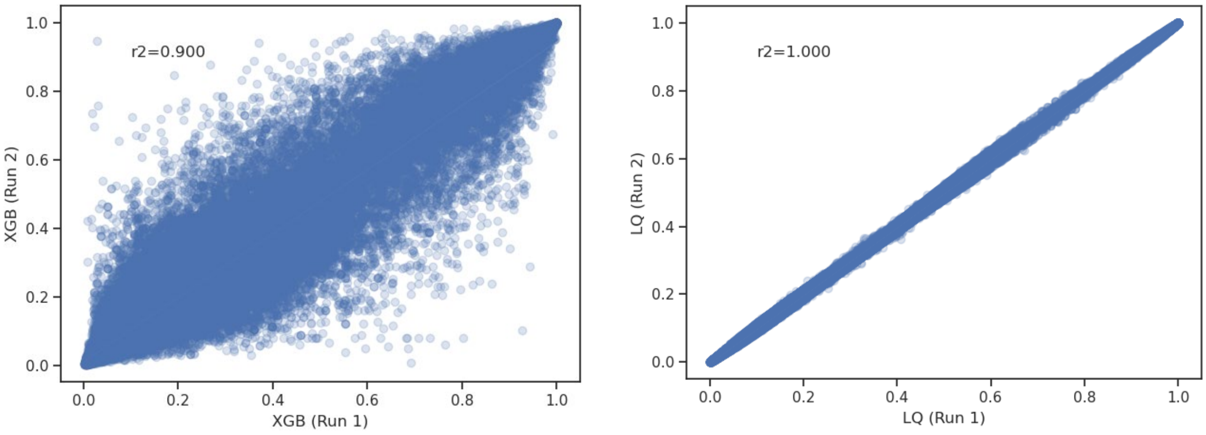

a **b**

Supplementary Fig. 9 Prediction correlation between two independent cross-validation model inferences. The predictions were collected from the leave-out fold predicted with a model trained based on the other four folds. **a** Results from two XGBoost models. **b** Results from two logistic regression *LQ* models.

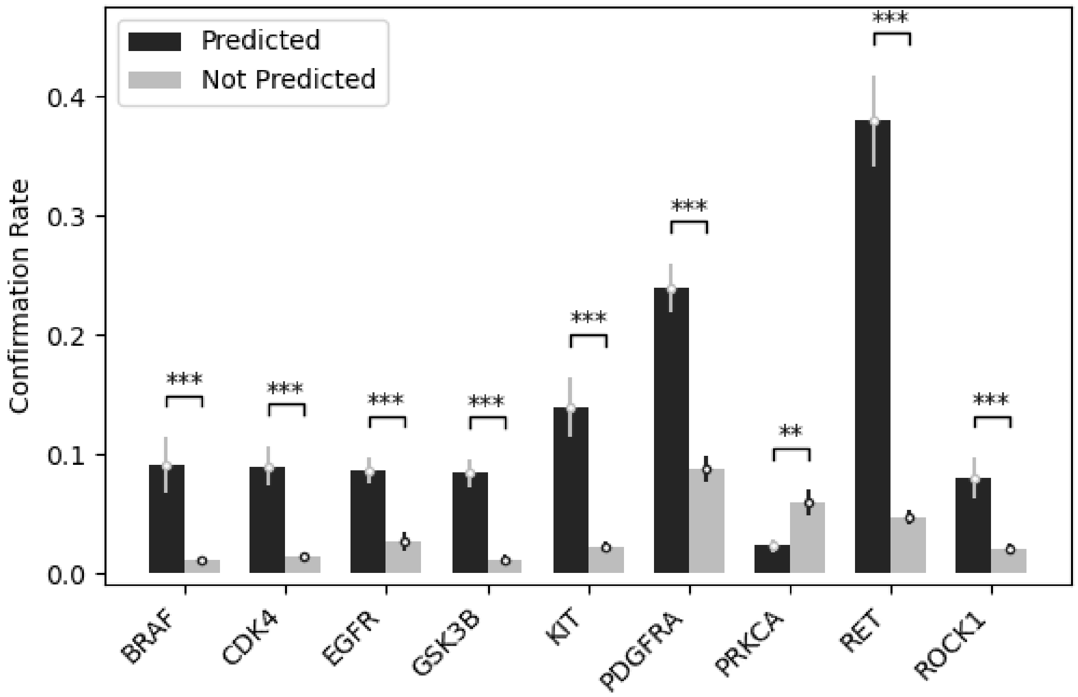

Supplementary Fig. 10 Comparison of dose-response confirmation rates between compounds predicted to bind to corresponding kinases (black) versus those not predicted to bind (gray). The differences show the precisions, TP/(TP + FP), of our kinase inhibitor predictions are significantly higher than their false omission rate, FN/(FN+TN). The separation is clear in 8 out of 9 assays tested except PRKCA. Error bars, mean ± SD. one-sided chi-square test. ** $p < 0.01$; *** $p < 0.001$. Statistics are in source data.

Supplementary Tables

Supplementary Table 1 The performance metrics of all compound target prediction models used in this study.

Note	Model	F1	MCC	TP	FP	TN	FN	Precision	Specificity	AUC	PCAUC
*	L	0.64	0.30	1449.00	738.87	1601.13	891.00	0.66	0.68	0.65	0.73
*	pQSAR (Q)	0.71	0.33	1911.00	1167.36	1172.64	429.00	0.62	0.50	0.66	0.87
*	Combined (LQ)	0.78	0.64	1605.00	148.43	2191.57	735.00	0.92	0.94	0.81	0.89
+	LQ_NB(Clip)	0.67	0.00	2340.00	2340.00	0.00	0.00	0.50	0.00	0.50	0.88
+	LQ_NB	0.76	0.49	1887.00	755.92	1584.08	453.00	0.71	0.68	0.74	0.87
#	NCI60	0.53	0.04	1261.00	1169.55	1170.45	1079.00	0.52	0.50	0.52	0.60
#	PSP	0.50	0.00	1165.00	1169.50	1170.50	1175.00	0.50	0.50	0.50	0.52
$	S (SAR)	0.62	0.22	1487.00	972.35	1367.65	853.00	0.60	0.58	0.61	0.81
@	L_NCI60	0.64	0.33	1410.00	644.56	1695.44	930.00	0.69	0.72	0.66	0.76
%	L_PSP	0.64	0.30	1444.00	732.36	1607.64	896.00	0.66	0.69	0.65	0.73

Notes

* Model L, Model pQSAR (a.k.a Model Q), and the combined model (a.k.a. Model LQ) were compared in Fig. S5a, where the integration of transcriptional data (L) and pharmacological data (Q) in Model LQ outperforms Model L and Model Q alone.
Model LQ is the best-performing model based on all metrics. Model S is not considered as its application is limited to predicting structural analogs of known reference compounds.

\+ Two forms of naive Bayesian (NB) models were compared with Model LQ in Fig. S8. Model LQ outperforms naive Bayesian models.

\# Model Q outperforms the models based on NCI60 data (Fig. S7) or in-house PSP data.

$ Models based on structural similarity. They are not adopted as their applications are limited to predicting structural analogs of known reference compounds.
Model SAR was trained based on a balanced in-house dataset; however, it is unable to capture remote structural analogs.
As we are interested in the ability of predicting compounds dissimilar to any reference compounds, Model SAR was only used for validation purpose instead of prediction.

@ Model L_NCI60 integrates transcriptional data (L) and pharmacological data as provided by the NCI60 dataset.

% Model L_PSP integrates transcriptional data (L) and pharmacological data as provided by the PSP dataset.

Supplementary Table 2 Confirmation rates and statistics of primary hits in 19 single-dose kinase assays grouped by compounds either predicted or not predicted to bind the corresponding target.

Target	Compounds Predicted to be Binders				Compounds Not Predicted to be Binders				P-value	Enrichment Factor
	Total Compounds Predicted	#Hits	Confirmation Rate (CR)	STDV of CR	Total Compounds Not Predicted	#Hits	Confirmation Rate (CR)	STDV of CR		
AURKA	156	39	0.25	0.035	960	37	0.039	0.0062	6.30E-22	6.5
BRAF	154	23	0.15	0.029	962	23	0.024	0.0049	8.80E-13	6.2
CDK2	389	42	0.11	0.016	727	12	0.017	0.0047	1.60E-11	6.5
CDK4	278	30	0.11	0.019	838	28	0.033	0.0062	1.30E-06	3.2
EGFR	677	127	0.19	0.015	439	23	0.052	0.011	8.90E-11	3.6
FLT3	305	89	0.29	0.026	811	53	0.065	0.0087	6.50E-24	4.5
GSK3B	501	57	0.11	0.014	615	12	0.02	0.0056	9.00E-11	5.8
IGF1R	178	27	0.15	0.027	938	19	0.02	0.0046	1.60E-15	7.5
INSR	200	33	0.17	0.026	916	17	0.019	0.0045	3.30E-19	8.9
KDR	403	70	0.17	0.019	713	9	0.013	0.0042	1.20E-23	14
KIT	202	43	0.21	0.029	914	75	0.082	0.0091	4.50E-08	2.6
LCK	562	80	0.14	0.015	554	21	0.038	0.0081	1.10E-09	3.8
LYN	117	54	0.46	0.046	999	49	0.049	0.0068	2.10E-47	9.4
MAPK14	489	32	0.065	0.011	627	9	0.014	0.0048	7.10E-06	4.6
PDGFRA	436	144	0.33	0.023	680	97	0.14	0.013	9.40E-14	2.3
PRKCA	658	25	0.038	0.0075	458	40	0.087	0.013	0.00043	0.44
RET	159	70	0.44	0.039	957	71	0.074	0.0085	1.80E-37	5.9
ROCK1	250	22	0.088	0.018	866	23	0.027	0.0055	1.50E-05	3.3
SRC	289	64	0.22	0.024	827	32	0.039	0.0067	2.30E-21	5.7

Statistics Footnotes

1 Column "Total Compounds Predicted" and "Total Compounds Not Predicted" contains sample counts for the corresponding bars in Fig 5a.

2 Columns "Confirmation Rate (CR)" contains the centers of the corresponding error bars in Fig 5a.

3 Columns "STDV of CR" contains the length of the error bars in Fig 5a.

4 Column "P-value" was calculated with one-sided chi-square test, single test.

Supplementary Table 3 The confirmation rates and statistics of secondary validation of selective primary hits in 9 dose-response kinase assays based on compounds either predicted or not predicted to bind the corresponding target.

Target	Compounds Single-dose Validated				Compounds Not Single-Dose Validated				P-value	Enrichment Factor
	Total Compounds Single-dose Validated	#Hits	Confirmation Rate (CR)	STDV of CR	Total Compounds Not Single-dose Validated	#Hits	Confirmation Rate (CR)	STDV of CR		
BRAF	44	25	0.57	0.075	301	10	0.033	0.01	4.50E-27	17.27
CDK4	56	37	0.66	0.063	291	46	0.16	0.021	1.40E-15	4.13
EGFR	147	70	0.48	0.041	255	6	0.024	0.0095	1.40E-28	20.00
GSK3B	66	49	0.74	0.054	281	10	0.036	0.011	2.90E-42	20.56
KIT	92	49	0.53	0.052	256	18	0.07	0.016	1.10E-21	7.57
PDGFRA	238	165	0.69	0.03	160	29	0.18	0.03	1.70E-23	3.83
PRKCA	65	42	0.65	0.059	280	50	0.18	0.023	2.70E-14	3.61
RET	139	105	0.76	0.036	210	36	0.17	0.026	2.30E-27	4.47
ROCK1	45	37	0.82	0.057	305	26	0.085	0.016	1.80E-32	9.65

Statistics Footnotes

1 Column "Total Compounds Predicted" and "Total Compounds Not Predicted" contains sample counts for the corresponding bars in Fig 5b.

2 Columns "Confirmation Rate (CR)" contains the centers of the corresponding error bars in Fig 5b.

3 Columns "STDV of CR" contains the length of the error bars in Fig 5b.

4 Column "P-value" was calculated with one-sided chi-square test, single test.

Supplementary Table 4 The fraction of gene signatures, where either RNA-seq embedding vector or Gene Ontology embedding vector dominates.

	Dominated* by GO	Dominated* by RNA-seq
**In Compound signatures**	1.29%	8.75%
**In shRNA signatures**	7.59%	18.87%
**In cDNA signatures**	2.17%	10.85%

* If 80% or more of the genes have weights in the linear combination determined by a single source, then that source dominates the weight values.

Supplementary References

1. Smaili, F. Z., Gao, X. & Hoehndorf, R. OPA2Vec: combining formal and informal content of biomedical ontologies to improve similarity-based prediction. *Bioinformatics* **35**, 2133–2140 (2019).

2. Du, J. *et al.* Gene2vec: distributed representation of genes based on co-expression. *BMC Genomics* **20**, 82 (2019).

3. Wang, S., Cho, H., Zhai, C., Berger, B. & Peng, J. Exploiting ontology graph for predicting sparsely annotated gene function. *Bioinformatics* **31**, i357-64 (2015).

4. Mikolov, T., Sutskever, I., Chen, K., Corrado, G. S. & Dean, J. Distributed representations of words and phrases and their compositionality. *Adv Neural Inf Process Syst* **26**, (2013).

5. Canese, K. & Weis, S. PubMed: The Bibliographic Database. *The NCBI handbook* **2**, (2013).

6. Zhong, F. *et al.* Drug target inference by mining transcriptional data using a novel graph convolutional network framework. *Protein Cell* **13**, 281–301 (2022).

7. Corsello, S. M. *et al.* The Drug Repurposing Hub: a next-generation drug library and information resource. *Nat Med* **23**, 405–408 (2017).

8. Madhukar, N. S. *et al.* A Bayesian machine learning approach for drug target identification using diverse data types. *Nat Commun* **10**, 5221 (2019).

9. Rogers, D. & Hahn, M. Extended-connectivity fingerprints. *J Chem Inf Model* **50**, 742–54 (2010).

10. Cheng, T., Li, Q., Wang, Y. & Bryant, S. H. Identifying compound-target associations by combining bioactivity profile similarity search and public databases mining. *J Chem Inf Model* **51**, 2440–8 (2011).

11. Martin, E. J. *et al.* All-Assay-Max2 pQSAR: Activity Predictions as Accurate as Four-Concentration IC50s for 8558 Novartis Assays. *J Chem Inf Model* **59**, 4450–4459 (2019).

12. Shoemaker, R. H. The NCI60 human tumour cell line anticancer drug screen. *Nat Rev Cancer* **6**, 813–23 (2006).

13. Matthews, B. W. Comparison of the predicted and observed secondary structure of T4 phage lysozyme. *Biochim Biophys Acta* **405**, 442–51 (1975).

14. Wang, W., Yang, S. & Li, J. Drug target predictions based on heterogeneous graph inference. *Pac Symp Biocomput* 53–64 (2013).

15. Zhou, Y. *et al.* Metascape provides a biologist-oriented resource for the analysis of systems-level datasets. *Nat Commun* **10**, 1523 (2019).

16. Chen, T. & Guestrin, C. XGBoost: A Scalable Tree Boosting System. *Proceedings of the 22nd ACM SIGKDD International Conference on Knowledge Discovery and Data Mining* doi:10.1145/2939672.

17. Pabon, N. A. *et al.* Predicting protein targets for drug-like compounds using transcriptomics. *PLoS Comput Biol* **14**, e1006651 (2018).

18. Noh, H., Shoemaker, J. E. & Gunawan, R. Network perturbation analysis of gene transcriptional profiles reveals protein targets and mechanism of action of drugs and influenza A viral infection. *Nucleic Acids Res* **46**, e34 (2018).

19. Lamb, J. *et al.* The Connectivity Map: using gene-expression signatures to connect small molecules, genes, and disease. *Science* **313**, 1929–35 (2006).
