# Supplementary material for: Drug target prediction through deep learning functional representation of gene signatures
Source: Nat Commun. 2024 Feb 29;15:1853. doi: 10.1038/s41467-024-46089-y (PMC10904399; doi:10.1038/s41467-024-46089-y)
Supplement: Supplementary file 3 — Description of Additional Supplementary Files [file 41467_2024_46089_MOESM3_ESM.pdf]

Drug target prediction through deep learning functional representation of gene signatures

Chen et al.

Supplementary Data Legends

File Name: “Supplementary Data 1. PredictedNetworkRelease.xlsx”
Description: 5133 pairs of compound-target predictions with multiple lines of validation evidence.

File Name: “Supplementary Data 2. KinaseHeatMap.xlsx”
Description: The IC_{50} values of 191 compounds across 9 kinase assays.

File Name: “Supplementary Data 3. AHR_DoseResponse_Confirmation.xlsx”
Description: The IC_{50} of 76 confirmed AhR binders in AhR agonist, antagonist, and toxicity assays.

File Name: “Supplementary Data 4. KinaseReactionConditionSummary.xlsx”
Description: Reaction conditions used in the kinase assays.
