# Supplementary material for: Drug target prediction through deep learning functional representation of gene signatures
Source: Nat Commun. 2024 Feb 29;15:1853. doi: 10.1038/s41467-024-46089-y (PMC10904399; doi:10.1038/s41467-024-46089-y)
Supplement: Supplementary file 8 — Reporting Summary [file 41467_2024_46089_MOESM8_ESM.pdf]

nature portfolio

Corresponding author(s): Hao Chen, Yingyao Zhou

Last updated by author(s): Feb 4, 2024

Reporting Summary

Nature Portfolio wishes to improve the reproducibility of the work that we publish. This form provides structure for consistency and transparency in reporting. For further information on Nature Portfolio policies, see our Editorial Policies and the Editorial Policy Checklist.

Statistics

For all statistical analyses, confirm that the following items are present in the figure legend, table legend, main text, or Methods section.

n/a	Confirmed	
☐	☒	The exact sample size (*n*) for each experimental group/condition, given as a discrete number and unit of measurement
☐	☒	A statement on whether measurements were taken from distinct samples or whether the same sample was measured repeatedly
☐	☒	The statistical test(s) used AND whether they are one- or two-sided<br>*Only common tests should be described solely by name; describe more complex techniques in the Methods section.*
☒	☐	A description of all covariates tested
☐	☒	A description of any assumptions or corrections, such as tests of normality and adjustment for multiple comparisons
☐	☒	A full description of the statistical parameters including central tendency (e.g. means) or other basic estimates (e.g. regression coefficient) AND variation (e.g. standard deviation) or associated estimates of uncertainty (e.g. confidence intervals)
☐	☒	For null hypothesis testing, the test statistic (e.g. *F*, *t*, *r*) with confidence intervals, effect sizes, degrees of freedom and *P* value noted<br>*Give P values as exact values whenever suitable.*
☒	☐	For Bayesian analysis, information on the choice of priors and Markov chain Monte Carlo settings
☒	☐	For hierarchical and complex designs, identification of the appropriate level for tests and full reporting of outcomes
☐	☒	Estimates of effect sizes (e.g. Cohen's *d*, Pearson's *r*), indicating how they were calculated

Our web collection on statistics for biologists contains articles on many of the points above.

Software and code

Policy information about availability of computer code

Data collection	The developer API of the Connectivity Map (https://clue.io) was utilized to extract Broad's compound-target annotations, while gene ontology data was processed with Metascape 3.5 (https://metascape.org). All data processing scripts were developed using Python 3 and relevant Python libraries.
Data analysis	Gene ontology enrichment analyses were carried out using Metascape 3.5 (https://metascape.org). CMAP scores were calculated with GSEA (https://www.gsea-msigdb.org/gsea). The data analysis scripts were written in Python 3. For deep learning models, TensorFlow 2.0 was utilized, with the code available at https://github.com/chenhcs/FRoGS.

For manuscripts utilizing custom algorithms or software that are central to the research but not yet described in published literature, software must be made available to editors and reviewers. We strongly encourage code deposition in a community repository (e.g. GitHub). See the Nature Portfolio guidelines for submitting code & software for further information.

Data

Policy information about availability of data

All manuscripts must include a data availability statement. This statement should provide the following information, where applicable:

- Accession codes, unique identifiers, or web links for publicly available datasets
- A description of any restrictions on data availability
- For clinical datasets or third party data, please ensure that the statement adheres to our policy

pQSAR activity dataset and PSP dataset are large-scale proprietary Novartis in-house resources, which cannot be released due to confidentiality restrictions. All other datasets were from the public domain. The level-5 perturbagen profiles for the L1000 Connectivity Map dataset were downloaded from the Gene Expression Omnibus (https://www.ncbi.nlm.nih.gov/geo) under Accession IDs GSE92742 [https://www.ncbi.nlm.nih.gov/geo/query/acc.cgi?acc=GSE92742] and GSE70138 [https://www.ncbi.nlm.nih.gov/geo/query/acc.cgi?acc=GSE70138]. The Broad's compound-target annotations were extracted from the Connectivity Map website (https://clue.io) using the developer API. The Reactome pathway data were downloaded from https://reactome.org. Gene Ontology data were downloaded from http://geneontology.org and processed by Metascape (https://metascape.org). ARCHS4 gene expression profiles were obtained from https://maayanlab.cloud/archs4 with the data file link https://s3.amazonaws.com/mssm-seq-matrix/human_matrix.h5. The US National Cancer Institute 60 human tumor cell line anticancer drug screen (NCI60) dataset were downloaded from https://discover.nci.nih.gov/cellminer. The experimental data for kinase binders and AhR binders can be found in Supplementary Table 2-3 and Supplementary Data 2-3. After removing 1163 compound-target pairs that are validated only according to in-house activity database, 5133 out of the 6296 predictions with multiple lines of additional validation evidence are made available as a community resource in Supplementary Data 1. Source data are provided with this paper.

Research involving human participants, their data, or biological material

Policy information about studies with human participants or human data. See also policy information about sex, gender (identity/presentation), and sexual orientation and race, ethnicity and racism.

Reporting on sex and gender	N/A
Reporting on race, ethnicity, or other socially relevant groupings	N/A
Population characteristics	N/A
Recruitment	N/A
Ethics oversight	N/A

Note that full information on the approval of the study protocol must also be provided in the manuscript.

Field-specific reporting

Please select the one below that is the best fit for your research. If you are not sure, read the appropriate sections before making your selection.

☒ Life sciences ☐ Behavioural & social sciences ☐ Ecological, evolutionary & environmental sciences

For a reference copy of the document with all sections, see nature.com/documents/nr-reporting-summary-flat.pdf

Life sciences study design

All studies must disclose on these points even when the disclosure is negative.

Sample size	All compounds predicted to bind to the selected kinase targets and AhR target, subjected to their availability in the Novartis compound store, were selected for experimental testing without triage. This includes a total of 1116 compounds predicted to bind to any of a set of 19 kinases and a total of 333 compounds predicted to bind to AhR.
Data exclusions	Compounds unavailable in the Novartis compound storage were not included.
Replication	Compounds predicted to be kinase inhibitors were profiled at 50uM in triplicate and primary hits were defined as active in at least two replicate. Primary hits were selected for an 8-point dose-response in duplicate and data points were pooled for analysis. Compounds predicted to bind to AhR were profiled in a single-dose in quadruplicate, and hits were selected for an 8-point dose-response in duplicate. All replications passed quality control.
Randomization	The goal of the biochemical assays was to follow-up on the the predictions made by FRoGS, thus specific the activity of each compound-target pair was measured and no comparison was made among compounds. The compound plating was randomized by the compound management group to our source plates, so the scientist was blinded to the identity of each compound-target pair until after data acquisition and analysis.
Blinding	The scientist who ran the biochemical assays was blinded to the identity of each compound-target pair until after data acquisition and analysis.

Reporting for specific materials, systems and methods

We require information from authors about some types of materials, experimental systems and methods used in many studies. Here, indicate whether each material, system or method listed is relevant to your study. If you are not sure if a list item applies to your research, read the appropriate section before selecting a response.

Materials & experimental systems

n/a	Involved in the study
☐	☒ Antibodies
☐	☒ Eukaryotic cell lines
☒	☐ Palaeontology and archaeology
☒	☐ Animals and other organisms
☒	☐ Clinical data
☒	☐ Dual use research of concern
☒	☐ Plants

Methods

n/a	Involved in the study
☒	☐ ChIP-seq
☒	☐ Flow cytometry
☒	☐ MRI-based neuroimaging

Antibodies

Antibodies used	Antibodies used in the kinase assays are described in Supplementary Table 5.
Validation	Most antibodies were commercially available from PerkinElmer (now Revvity), with the exception of phospho-(ser) 14-3-3 binding motif mouse mAb and phospho-Rb (Ser780) rabbit mAb, which were sourced from Cell Signaling Technology (see Supplement Table 5). All antibodies exhibited selective reactivity with the corresponding phosphorylated substrate over the unphosphorylated substrate in the biochemical assays using purified enzyme.

Eukaryotic cell lines

Policy information about cell lines and Sex and Gender in Research

Cell line source(s)	The cells are engineered from the human HepG2 hepatoma cell line for the study of the aryl hydrocarbon receptor (AhR) genomic signaling induction by monitoring the activity of the Lucia luciferase report protein.
Authentication	HepG2-Lucia AhR cells were purchased from InvivoGen (San Diego, CA). The catelog code is hpgl-ahr. More details can be found at https://www.invivogen.com.
Mycoplasma contamination	According to Invivogen, HepG2-Lucia AhR cells are guaranteed mycoplasma-free.
Commonly misidentified lines (See ICLAC register)	Not applicable.
